# Pressure‐Induced Structural Evolution and Bandgap Optimization of Lead‐Free Halide Double Perovskite (NH_4_)_2_SeBr_6_

**DOI:** 10.1002/advs.201902900

**Published:** 2020-01-27

**Authors:** Lingrui Wang, Panpan Yao, Fei Wang, Shunfang Li, Yaping Chen, Tianyu Xia, Erjia Guo, Kai Wang, Bo Zou, Haizhong Guo

**Affiliations:** ^1^ Key Laboratory of Materials Physics of Ministry of Education School of Physics Zhengzhou University Zhengzhou 450001 China; ^2^ International Laboratory for Quantum Functional Materials of Henan School of Physics Zhengzhou University Zhengzhou 450001 China; ^3^ State Key Laboratory of Superhard Materials Jilin University Changchun 130012 China; ^4^ Beijing National Laboratory for Condensed Matter Physics and Institute of Physics Chinese Academy of Sciences Beijing 100190 China; ^5^ Collaborative Innovation Center of Light Manipulations and Applications Shandong Normal University Jinan 250358 China

**Keywords:** diamond anvil cells, double perovskites, optical properties, reorganization

## Abstract

Lead‐free halide double perovskites (HDPs) are promising candidates for high‐performance solar cells because of their environmentally‐friendly property and chemical stability in air. The power conversion efficiency of HDPs‐based solar cells needs to be further improved before their commercialization in the market. It requires a thoughtful understanding of the correlation between their specific structure and property. Here, the structural and optical properties of an important HDP‐based (NH_4_)_2_SeBr_6_ are investigated under high pressure. A dramatic piezochromism is found with the increase in pressure. Optical absorption spectra reveal the pressure‐induced red‐shift in bandgap with two distinct anomalies at 6.57 and 11.18 GPa, and the energy tunability reaches 360 meV within 20.02 GPa. Combined with structural characterizations, Raman and infrared spectra, and theoretical calculations using density functional theory, results reveal that, the first anomaly is caused by the formation of a Br‐Br bond among the [SeBr_6_]^2−^ octahedra, and the latter is attributed to a cubic‐to‐tetragonal phase transition. These results provide a clear correlation between the chemical bonding and optical properties of (NH_4_)_2_SeBr_6_. It is believed that the proposed strategy paves the way to optimize the optoelectronic properties of HDPs and further stimulate the development of next‐generation clear energy based on HDPs solar cells.

## Graphical abstract

Pressure‐induced optical properties variation of lead‐free halide double perovskite (NH_4_)_2_SeBr_6_ is strongly correlated with the re‐organization of Br‐Br bonds and the rotation and distortion of [SeBr_6_]^2−^ octahedra. The structure–optical property relationship constructed here is a straightforward approach to optimize the optoelectronic properties of halide double perovskites (HDPs) and further stimulate the development of next‐generation clear energy based on HDPs solar cells.

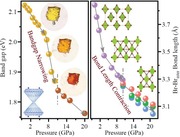

## 1 Introduction

Organic–inorganic lead (Pb) halide perovskite solar cells have attracted a tremendous amount of attentions because of their remarkable power conversion efficiency (PCE), which has increased from 3.8% in 2009 to 25.2% in 2019.^1^, ^2^ However, the commercialization of this photovoltaic technology still faces two major concerns, i.e., Pb toxicity and material instability.^3^, ^4^, ^5^ Therefore, finding alternative, non‐ or low‐toxicity, and stable halide perovskites for optimization of the performance of perovskites solar cells is a great challenge. Among various strategies, the heterovalent substitution concept is invoked to design/fabricate A_2_BX_6_ lead‐free halide double perovskite (HDP) structure, where a pair of Pb(II) ions is replaced by one tetravalent B^4+^ ion. Based on such approach, various relatively more stable and nontoxic HDP materials have been proposed.^6^, ^7^, ^8^, ^9^, ^10^

The A_2_BX_6_ HDP structure, which is characterized by isolated [BX_6_]^2−^ octahedral units bridged with A‐site cations, is in contrast to the corner‐sharing arrangement of the ABX_3_ perovskite species. Associated with this structural change, Cs_2_SnI_6_ is reported to exhibit higher stability than CsSnI_3_ because of its stronger Sn‐I bonding due to the shortened Sn‐I bond lengths in the [SnI_6_]^2−^ octahedral units. As a consequence, such a bonding change within the octahedral units significantly affected the boundary conditions of electronic wave functions and led to the reduced bandgap of the former as compared with the latter.^8^, ^11^ Moreover, the optoelectronic properties can be further tuned by replacing the Cs^+^ cation on the A site with an organic ligand such as NH_4_^+^ or CH_3_NH_3_^+^. Specifically, the electronic structure can be effectively modified via coupling the ligands to the rotational dynamics of [BX_6_]^2−^ octahedral units through the enhanced hydrogen‐bonding interactions with the surrounding X‐site framework.^12^, ^13^ Clearly, such findings demonstrate that the optical properties of HDPs are strongly related to the specific crystal structures. (NH_4_)_2_SeBr_6_ is a typical HDP material that is capable of tuning chemical and physical properties by modifying its structure, and thus it is needed to be investigated further.

Pressure is a controllable and predictable way that is extensively used to precisely tune the properties of various materials.^14^, ^15^, ^16^ For example, we have experimentally investigated the high‐pressure effect on the properties of organic–inorganic lead halide perovskite CH(NH_2_)_2_PbBr_3_, and found that its structure undergoes two phase transitions below 2.2 GPa, directly leading to the red shift/blue shift of bandgap and the variation of the colors.^17^ Strikingly, upon applying pressure of up to 2.1 GPa, the bandgap of CH(NH_2_)_2_PbI_3_ is reported to experience a red shift from 1.489 to 1.337 eV, which exactly reaches to the optimal bandgap required by Shockley–Queisser efficiency limit.^18^ Meanwhile, Jia et al. found that CH_3_NH_3_SnI_3_ can possess structural stability, increased electrical conductivity, and enhanced photo responsiveness via two sequential compression and decompression cycles.^19^ Such efforts on tailoring the properties of organic–inorganic lead halide perovskites via the introduction of pressure are constructive to meet the requirement of practical application in optoelectronic devices. Yet, up to date, it is still an open area in simultaneously tuning the intra‐octahedral B‐X bonding and the inter‐octahedral X‐X interactions to improve the PCE of A_2_BX_6_ HDPs by introducing pressure. Moreover, these high‐pressure effects of structural modifications on the bandgap of HDPs can be reproduced by chemical‐pressure and/or the strain induced by a substrate on thin films for photovoltaic applications.

Here, we adopted symmetric diamond anvil cell high pressure techniques, including UV‐vis absorption, in situ angle‐dispersive X‐ray diffraction (ADXRD), Raman and IR measurements, as well as density functional theory (DFT) calculations to map the pressure effect on the structure‐property relationship of an important HDP‐based (NH_4_)_2_SeBr_6_. Dramatic piezochromism varying from initial light‐yellow to red optical absorption spectra reveal the pressure‐induced red‐shift in the bandgap with two distinct anomalies at 6.57 and 11.18 GPa and the energy tunability ranges up to 360 meV within 20.02 GPa. DFT calculations further convincingly reveal that, the first anomaly is caused by the formation of Br‐Br bonds of the inter‐[SeBr_6_]^2‐^ octahedra; and the latter is attributed to a cubic‐to‐tetragonal phase transition with the rotation and distortion of [SeBr_6_]^2−^ octahedra driven by the pressure, which is accompanied with lifting of the degeneracy of the Se‐Br bonds in the aid of hydrogen bonding. The present findings not only establish the underlying microspectroscopic mechanism of the pressure effect on tuning the specific atomic structure and thus the optical properties of (NH_4_)_2_SeBr_6_ at high pressure, but are also expected to present a straightforward approach for the design of new HDPs with superior properties and offer the possibility of new opportunities and strategies for practical photovoltaic applications.

## 2 Results and Discussion

**Figure 1:**
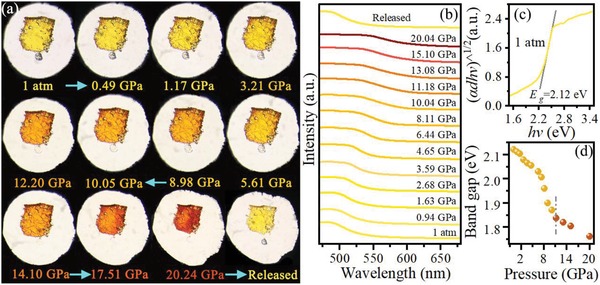
a) Optical micrographs of the piezochromic phenomenon. b–d) Selected optical absorption spectra, indirect bandgap Tauc plot under ambient conditions and bandgap evolutions of (NH_4_)_2_SeBr_6_ as a function of pressure, respectively. The vertical dashed line represents the possible phase transition pressure.

First, we investigate the pressure effects on the optical properties of (NH_4_)_2_SeBr_6_, which could shed light on understanding the electronic structure and characteristic of the bandgaps. Under ambient conditions, (NH_4_)_2_SeBr_6_ displays light yellow color. With increased pressure, we observe piezochromism as manifested in the color changes, i.e., the light‐yellow color gradually changes to red up to 20.24 GPa (**Figure** **1a**). The red shifts of the optical absorption edge as a function of pressure are presented in Figure 1b. Based on the results of the optical measurements, the bandgap of (NH_4_)_2_SeBr_6_ is estimated to 2.12 eV under ambient conditions (Figure 1c). However, the bandgap continuously decreases to 1.76 eV at 20.02 GPa, showing the powerful ability of pressure on tailoring the bandgap of (NH_4_)_2_SeBr_6_. During this pressure range, the bandgap narrows about 360 meV in the visible spectrum within 20.02 GPa. The sensitivity of bandgap of this material under pressure would provide an applicable strategy for the fabrication of (NH_4_)_2_SeBr_6_‐based sensor and switcher devices. Moreover, the change in the bandgap as a function of pressure presents two turning points at 6.57 and 11.18 GPa, respectively, as shown in Figure 1d. Considering that the bandgap is determined by their crystalline structure, the two discontinuities in the bandgap may be related to the structural transitions.^20^, ^21^ After releasing the pressure, the absorption of optical color and spectrum revert to their original state, which indicates reversible changes of the structures. These results clearly demonstrate that, the modulation of the optical properties of (NH_4_)_2_SeBr_6_ can be achieved by the application of pressure, which could further optimize its performance in solar energy conversion application.

**Figure 2:**
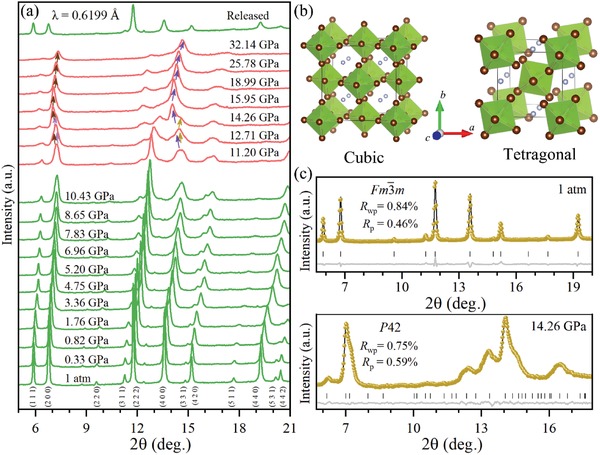
a) High‐pressure evolution of the ADXRD patterns of (NH_4_)_2_SeBr_6_ at various pressures. b) Crystal structure of (NH_4_)_2_SeBr_6_ at cubic phase (*Fm*‐3*m*) and tetragonal phase (*P*42). c) Rietveld refinements of (NH_4_)_2_SeBr_6_ crystal at ambient pressure (cubic phase) and at 14.26 GPa (tetragonal phase).

Structural variation is believed to be responsible for the intriguing color changes and the bandgap shifts. **Figure** **2**a shows the typical ADXRD patterns of the (NH_4_)_2_SeBr_6_ samples obtained at various pressures during compression of up to 32.14 GPa and decompression. The XRD pattern under ambient condition reveals that the initial crystal structure is the pure cubic phase with space group *Fm*‐3*m*. The cubic structure of (NH_4_)_2_SeBr_6_ crystal is used as a starting pattern, and the refinement results in cell parameter *a* = 10.4826 Å (Figure 2b‐c).^22^ As pressure increases, all diffraction peaks show unobvious changes except for shifts to higher angles, indicating the stabilization and sustaining contraction of the cubic phase. Significant IR spectra of N‐H bending and N‐H stretching made red shift occur simultaneously (Figure S1‐S2, Supporting Information). Since the atom H in the N‐H group is more electropositive than N, the electrostatic attraction between H and Br increases, from the correlation, this should lead to an increase in the N‐H bond length and reducing its restoring force and stretching frequency. Namely, the red shifts are attributed to the strengthened hydrogen bonds at high pressures.^23^, ^24^ The situation is quite similar to water and ice under compression and base solvation in which anion–anion repulsion comes into play.^25^ The XRD pattern dramatically changes at 11.20 GPa, including the splitting of two peaks at 7.2° and 14.6° and the shifting to lower angles (indicated by arrows). All of these changes confirm that (NH_4_)_2_SeBr_6_ undergoes a symmetry‐breaking phase transition at 11.20 GPa. Additional splitting of the Se‐Br bending and stretching modes are also observed in Raman spectra at 11.05 GPa (Figure S1, Supporting Information), directly supporting the phase transition of (NH_4_)_2_SeBr_6_ from high‐symmetry to low‐symmetry structure.^26^, ^27^ Meanwhile, the N‐H bending mode split into two modes at 11.98 GPa, also providing the indirect evidence of the rotation and distortion of the [SeBr_6_]^2−^ octahedra due to the hydrogen bond interaction. Figure 2b and Figure S3–S4 (Supporting Information) show Rietveld refinements of (NH_4_)_2_SeBr_6_ crystal at 14.26 GPa, and a tetragonal with space group *P*42 is used to fit the split diffraction peaks. The structural analysis shows that the phase transition corresponds to two bond lengths for (NH_4_)_2_SeBr_6_, and an angle rotation for the [SeBr_6_]^2−^ inorganic octahedra relative to the cubic axes. Namely, the structural phase transition from cubic to tetragonal phase is characterized by the rotation and distortion of [SeBr_6_]^2−^ octahedra. With the pressure increasing, the XRD peaks subsequently shift toward higher angles and become slightly broader. After complete pressure releases, the phase transition observed is reversible, as evidenced by the return of the diffraction pattern to its initial states. And the flexible organic cations NH_4_^+^ serve as templates in [SeBr_6_]^2−^ octahedral frameworks are responsible for the structural memory effect in reversible pressure‐induced phase transition.

**Figure 3:**
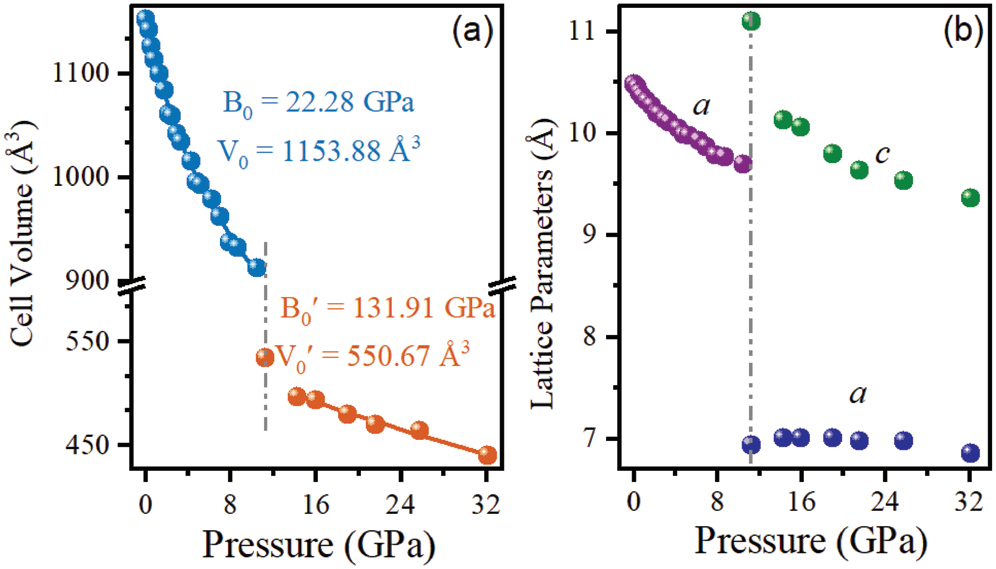
High‐pressure evolution of a) the unit‐cell volume and b) lattice parameters of (NH_4_)_2_SeBr_6_. Dash lines represent the phase transition pressure.

To further obtain insights on the structural phase transition, the variation of the lattice parameters and unit‐cell volume of (NH_4_)_2_SeBr_6_ as a function of pressure is plotted, as shown in **Figure** **3**a. As expected, volume decreases gradually with increased pressure during compression (Figure 3a and Figure S5, Supporting Information). The calculated bulk moduli are: *B*_0_ = 22.28 GPa for the cubic phase and *B*_0_′ = 131.91 GPa for the tetragonal phase. These values are estimated by utilizing the third‐order Birch–Murnaghan equation of state.^28^ The high value of bulk moduli implies a more robust structure of tetragonal (NH_4_)_2_SeBr_6_, which may be propitious to enhance the stability of solar cells. As shown in Figure 3b, the phase transition at 11.20 GPa is also characterized by considerable changes in the lattice parameters (*a, c*). Specifically, the tetragonal (NH_4_)_2_SeBr_6_ shows an anisotropic compressibility with its *c* parameter appearing more compressible than *a* and *b* in the *ab* plane. These behaviors reveal that the phase transition is a first‐order type, and the rotation of the [SeBr_6_]^2−^ octahedra is responsible for the phase transformation of (NH_4_)_2_SeBr_6_ under pressure.

**Figure 4:**
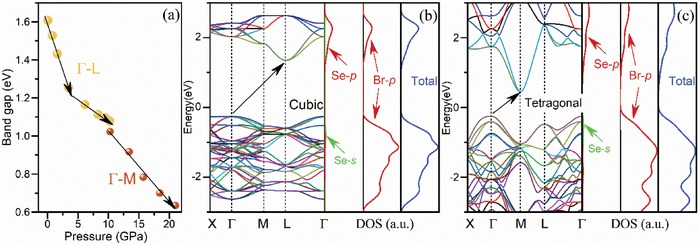
a) DFT calculated bandgap under different pressures. b,c) Calculated electronic band structures and projected density of states for (NH_4_)_2_SeBr_6_ at cubic and tetragonal phase (1 atm and 14.26 GPa in experiment).

As suggested, the bandgap of perovskite materials is highly variable based on the overlap of electronic wave functions between metal B and halide X ions.^29^, ^30^, ^31^, ^32^, ^33^, ^34^ **Figure** **4**a shows the first‐principles DFT calculated bandgap versus pressure. Considering the well‐known issue of the underestimation of the bandgap in plain DFT calculations, the calculated bandgap variation curve is overall in good consistency with the experimental results. Especially, the calculated two discontinuity points are well consistent with the experimental results (as shown in Figure 1d). Specifically, first‐principles calculations show that the bandgap of (NH_4_)_2_SeBr_6_ changes from being indirect (Γ–L) to indirect (Γ–M) with the phase transformation from cubic to tetragonal, as shown in Figure 4b–c. The valence band maximum (VBM) is effectively pinned to the nonbonding Br 4*p* states which hybridize slightly with the Se 4*s* states, while the conduction band minimum (CBM) is characterized as the antibonding hybrid state of the Se 4*p* and Br 4*p* orbitals. When pressure is applied to the cubic phase of (NH_4_)_2_SeBr_6_, the [SeBr_6_]^2−^ octahedra are contracted and the bond length of Se‐Br decreases. As a result, CBM elevates to higher energy regime under pressure due to its antibonding characteristics. Meanwhile, the overlap between nonbonding Br *p* orbitals increases, resulting in a large broadening of the highest valence band. Given the stiffness of the octahedra, the Se‐Br bond is considerably stronger than the Br‐Br bond either on the surface of the octahedra (named as intra‐octahedra Br‐Br bond) or between the isolated octahedra (named as inter‐octahedra Br‐Br bond). Hence, VBM rises up greater that of the CBM, and consequently the bandgap is narrowed (as shown in the projected density of states of Figure 4b–c, and Figure S6, Supporting Information). Moreover, the X‐X bond distances may also play an important role in tuning the bandgap of the A_2_BX_6_ perovskites.^35^, ^36^, ^37^ Our experimental results and findings are detailed as follows.

**Figure 5:**
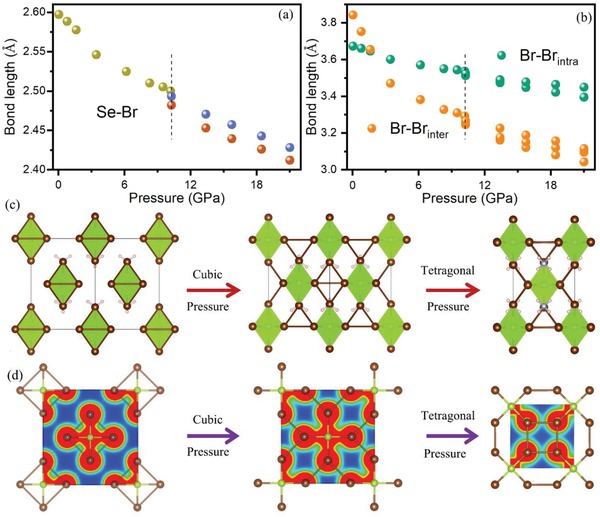
DFT calculated bond length for a) Se‐Br and b) Br‐Br under different pressures. c) Schematic illustrations of the neighboring Br‐Br bonds and d) charge density isosurface of the (001) surface at cubic phase (1 atm and mild pressure), and tetragonal phase (high pressure). The neighboring Br‐Br bonds are illustrated as the brown stick.

Now, we discuss the pressure‐induced Br‐Br bond evaluation in (NH_4_)_2_SeBr_6_. As shown in **Figure** **5**a, significantly, the Se‐Br bond lengths are split into two values after the cubic‐to‐tetragonal phase transition. This finding indicates that a symmetry breaking occurs within the [SeBr_6_]^2−^ octahedra, i.e., the rotation of the [SeBr_6_]^2−^ octahedra is accompanied with a distortion of the [SeBr_6_]^2‐^ octahedra, which is responsible for the phase transformation of (NH_4_)_2_SeBr_6_ under pressure. Figure 5b presents the Br‐Br bond lengths under various pressures. Two distinct Br‐Br bond lengths are observed before the phase transition, and increases to five after the phase transition. Specifically, the intra‐octahedral Br‐Br bonds divide into two groups of unequal bonds due to the distortion of [SeBr_6_]^2−^ octahedra as the octahedra elongate slightly in the *c* direction. Moreover, the inter‐octahedral Br‐Br bond lengths are changed to three cases, due to the rotation and distortion of the [SeBr_6_]^2−^ octahedra. For these three cases of Br‐Br bonds, one lies in the (001) surface, and the other two are the tilt Br‐Br bonds linking the isolated octahedra. Interestingly, we find that a crossover exists between the intra‐ and inter‐octahedral Br‐Br bond lengths in the cubic phase under about 3.50 GPa. The pressure‐induced bond length variation matches well with the discontinuity bandgap in Figure 4a.

To show the change in the bond length evolution more clearly, schematic illustrations of the neighboring Br‐Br bonds in cubic phase (1 atm and mild pressure), and tetragonal phase (high pressure) are presented in Figure 5c. Evidently, at 1 atm, the shortest Br‐Br bonds are located on the surface of each octahedral unit, termed as intra‐octahedral Br‐Br bonds. However, under mild pressure, approximately 3.50 GPa, the shortest Br‐Br bonds are characterized by the inter‐octahedral Br‐Br bonds which link the octahedral units. The mechanism of this bond length change can be readily understood from the viewpoint of the stiffness of the octahedral. Without pressure, the intra‐octahedral Br‐Br bond is considerably stronger than that of the inter‐octahedral Br‐Br bond due to the shorter Br‐Br bond length of the former as compared with that of the latter. Under pressure, the inter‐octahedral Br‐Br bond length is more readily changed compared with that of intra‐octahedral Br‐Br bond length due to the relatively weak van der Waals feature of the former Br‐Br bonds. With increased pressure, the shortest Br‐Br bonds of the cubic phase will definitely change from the intra‐ to inter‐octahedra under mild pressure of about 3.50 GPa in (NH_4_)_2_SeBr_6_. This cross point matches well with the first discontinuity bandgap presented in Figure 4a. With further increased pressure up to 10.20 GPa, as driven by the further enhanced inter‐octahedral Br‐Br bonds, the [SeBr_6_]^2−^ octahedra units are rotated and distorted. As a consequence, cubic‐to‐tetragonal phase transition occurs. Meanwhile, in such phase transition process, the symmetry breaking of the [SeBr_6_]^2−^ octahedral units as manifested by the tilting and elongation can further reduce the density of states by the Fermi surface and thus lower the total energy. This transition point matches well with the second discontinuity in the bandgap reduction. The above findings are further validated by charge density analysis. In Figure 5d, we show the charge density iso‐surfaces within the (001) plane including the shortest Br‐Br bonds of the cubic phase (1 atm and mild pressure) and tetragonal phase (high pressure). Clearly, the inter‐octahedral Br‐Br bond becomes stronger with the increased pressure. Therefore, collectively, the combined experiment and DFT calculations establish that the electronic structure variation of (NH_4_)_2_SeBr_6_ is strongly correlated with the re‐organization of Br‐Br bonds under pressure. These findings are expected to present a straightforward approach in optimizing the optoelectronic properties of related HDPs.

## 3 Conclusion

In summary, we establish the microspectroscopic mechanism of the pressure induced structural evolution and optical optimization of HDP‐based (NH_4_)_2_SeBr_6_. We found a dramatic piezochromism varying from initial light‐yellow to red as increasing the degree of lattice distortion by pressure. Our combined experiments and DFT calculations establish that the re‐organization of Br‐Br bonds and the rotation and distortion of [SeBr_6_]^2−^ octahedra are responsible for the bandgap anomalies at 6.57 and 11.18 GPa. These results provide a clear correlation between the chemical bonding and optical properties of (NH_4_)_2_SeBr_6_, and the universal role of structure induced bandgap alignment should be applied to other HDPs and further to understand, control, and design new HDPs and highlight their potential application for photovoltaic devices.

## Conflict of Interest

The authors declare no conflict of interest.

## Supporting information

Supporting Information

## References

[1] A. Kojima , K. Teshima , Y. Shirai , T. Miyasaka , J. Am. Chem. Soc. 2009, 131, 6050. 19366264 10.1021/ja809598r

[2] Best Research‐Cell Efficiency Chart , https://www.nrel.gov/pv/cellefficiency.html (accessed: November 2019).

[3] A. Abate , Joule 2017, 1, 659.

[4] X. Qin , Z. Zhao , Y. Wang , J. Wu , Q. Jiang , J. You , J. Semicond. 2017, 38, 011002.

[5] Q. Zhang , F. Hao , J. Li , Y. Zhou , Y. Wei , H. Lin , Sci. Technol. Adv. Mater. 2018, 19, 425. 29868147 10.1080/14686996.2018.1460176 PMC5974705

[6] X.‐G. Zhao , D. Yang , J.‐C. Ren , Y. Sun , Z. Xiao , L. Zhang , Joule 2018, 2, 1662.

[7] M.‐G. Ju , M. Chen , Y. Zhou , H. F. Garces , J. Dai , L. Ma , N. P. Padture , X. C. Zeng , ACS Energy Lett. 2018, 3, 297.

[8] X. Qiu , B. Cao , S. Yuan , X. Chen , Z. Qiu , Y. Jiang , Q. Ye , H. Wang , H. Zeng , J. Liu , M. G. Kanatzidis , Sol. Energy Mater. Sol. Cells 2017, 159, 227.

[9] L. Chu , W. Ahmad , W. Liu , J. Yang , R. Zhang , Y. Sun , J. Yang , X. a. Li , Nano‐Micro Lett. 2019, 11, 16. 10.1007/s40820-019-0244-6 PMC7770810 34137969

[10] A. E. Maughan , A. M. Ganose , D. O. Scanlon , J. R. Neilson , Chem. Mater. 2019, 31, 1184.

[11] Z. Xiao , H. Lei , X. Zhang , Y. Zhou , H. Hosono , T. Kamiya , Bull. Chem. Soc. Jpn. 2015, 88, 1250.

[12] A. E. Maughan , A. M. Ganose , A. M. Candia , J. T. Granger , D. O. Scanlon , J. R. Neilson , Chem. Mater. 2018, 30, 472.

[13] H. A. Evans , D. H. Fabini , J. L. Andrews , M. Koerner , M. B. Preefer , G. Wu , F. Wudl , A. K. Cheetham , R. Seshadri , Inorg. Chem. 2018, 57, 10375. 30074384 10.1021/acs.inorgchem.8b01597

[14] Y. Liang , X. Huang , Y. Huang , X. Wang , F. Li , Y. Wang , F. Tian , B. Liu , Z. X. Shen , T. Cui , Adv. Sci. 2019, 6, 1900399. 10.1002/advs.201900399 PMC6661939 31380210

[15] T. Yin , B. Liu , J. Yan , Y. Fang , M. Chen , W. K. Chong , S. Jiang , J.‐L. Kuo , J. Fang , P. Liang , S. Wei , K. P. Loh , T. C. Sum , T. J. White , Z. X. Shen , J. Am. Chem. Soc. 2019, 141, 1235. 30561996 10.1021/jacs.8b07765

[16] a) Z. Ma , Z. Liu , S. Lu , L. Wang , X. Feng , D. Yang , K. Wang , G. Xiao , L. Zhang , S. A. T. Redfern , B. Zou , Nat. Commun. 2018, 9, 4506; 30374042 10.1038/s41467-018-06840-8 PMC6206024

[17] L. Wang , K. Wang , B. Zou , J. Phys. Chem. Lett. 2016, 7, 2556. 27321024 10.1021/acs.jpclett.6b00999

[18] G. Liu , L. Kong , J. Gong , W. Yang , H.‐K. Mao , Q. Hu , Z. Liu , R. D. Schaller , D. Zhang , T. Xu , Adv. Funct. Mater. 2017, 27, 1604208.

[19] X. Lü , Y. Wang , C. C. Stoumpos , Q. Hu , X. Guo , H. Chen , L. Yang , J. S. Smith , W. Yang , Y. Zhao , H. Xu , M. G. Kanatzidis , Q. Jia , Adv. Mater. 2016, 28, 8663. 27514760 10.1002/adma.201600771

[20] a) G. Liu , L. Kong , W. Yang , H.‐K. Mao , Mater. Today 2019, 27, 91;

[21] A. Jaffe , Y. Lin , H. I. Karunadasa , ACS Energy Lett. 2017, 2, 1549.

[22] L. Sieg , Z. Anorg. Allg. Chem. 1932, 207, 93.

[23] B. V. S Murthy , K. P. Ramesh , J. Ramakrishna , Phase Transit. 1994, 46, 229.

[24] D. Liu , W. Lei , K. Wang , G. Bao , F. Li , J. Hao , B. Liu , T. Cui , Q. Cui , G. Zou , J. Phys. Chem. B 2009, 113, 7430. 19344166 10.1021/jp900467z

[25] a) C. Q. Sun , X. Zhang , W. Zheng , Chem. Sci. 2012, 3, 1455;

[26] P. J. Hendra , Z. Jović , J. Chem. Soc. A 1968, 600.

[27] E. R. Clark , M. A. Al‐Turaihi , J. Organomet. Chem. 1977, 124, 391.

[28] F. Birch , J. Geophys. Res. 1978, 83, 1257.

[29] L. Kong , G. Liu , J. Gong , Q. Hu , R. D. Schaller , P. Dera , D. Zhang , Z. Liu , W. Yang , K. Zhu , Y. Tang , C. Wang , S.‐H. Wei , T. Xu , H.‐K. Mao , Proc. Natl. Acad. Sci. USA 2016, 113, 8910. 27444014 10.1073/pnas.1609030113 PMC4987786

[30] S. Jiang , Y. Fang , R. Li , H. Xiao , J. Crowley , C. Wang , T. J. White , W. A. Goddard , Z. Wang , T. Baikie , F. Jangiye , Angew. Chem., Int. Ed. 2016, 55, 6540. 10.1002/anie.201601788 27101324

[31] Y. Wang , X. Lü , W. Yang , T. Wen , L. Yang , X. Ren , L. Wang , Z. Lin , Y. Zhao , J. Am. Chem. Soc. 2015, 137, 11144. 26284441 10.1021/jacs.5b06346

[32] A. Kaltzoglou , M. Antoniadou , A. G. Kontos , C. C. Stoumpos , D. Perganti , E. Siranidi , V. Raptis , K. Trohidou , V. Psycharis , M. G. Kanatzidis , P. Falaras , J. Phys. Chem. C 2016, 120, 11777.

[33] L. Zhang , C. Liu , L. Wang , C. Liu , K. Wang , B. Zou , Angew. Chem., Int. Ed. 2018, 57, 11213. 10.1002/anie.201804310 30010235

[34] Y. Huang , L. Wang , Z. Ma , F. Wang , J. Phys. Chem. C 2019, 123, 739.

[35] G. Bounos , M. Karnachoriti , A. G. Kontos , C. C. Stoumpos , L. Tsetseris , A. Kaltzoglou , X. Guo , X. Lü , Y. S. Raptis , M. G. Kanatzidis , P. Falaras , J. Phys. Chem. C 2018, 122, 24004.

[36] Y. Cai , W. Xie , H. Ding , Y. Chen , K. Thirumal , L. H. Wong , N. Mathews , S. G. Mhaisalkar , M. Sherburne , M. Asta , Chem. Mater. 2017, 29, 7740.

[37] M. G. Brik , I. V. Kityk , J. Phys. Chem. Solids 2011, 72, 1256.

