# Supplementary material for: Pressure‐Induced Structural Evolution and Bandgap Optimization of Lead‐Free Halide Double Perovskite (NH4)2SeBr6
Source: Adv Sci (Weinh). 2020 Jan 27;7(6):1902900. doi: 10.1002/advs.201902900 (PMC7080510; doi:10.1002/advs.201902900)
Supplement: Supplementary file 1 — Supporting Information [file ADVS-7-1902900-s001.pdf]

Copyright WILEY-VCH Verlag GmbH & Co. KGaA, 69469 Weinheim, Germany, 2020.

ADVANCED SCIENCE Open Access

Supporting Information

for *Adv. Sci.,* DOI: 10.1002/advs.201902900

Pressure-Induced Structural Evolution and Bandgap Optimization of Lead-Free Halide Double Perovskite $(NH_4)_2SeBr_6$

Lingrui Wang, Panpan Yao, Fei Wang, Shunfang Li, Yaping Chen, Tianyu Xia, Erjia Guo, Kai Wang,* Bo Zou, and Haizhong Guo**

Pressure-Induced Structural Evolution and Bandgap Optimization of Lead-Free Halide Double Perovskite $(NH_4)_2SeBr_6$

Lingrui Wang, Panpan Yao, Fei Wang, Shunfang Li, Yaping Chen, Tianyu Xia, Erjia Guo, Kai Wang*, Bo Zou, and Haizhong Guo**

*Corresponding author. Email: wfei@zzu.edu.cn; kaiwang@jlu.edu.cn; hguo@zzu.edu.cn;

This PDF file includes：

Experimental Section
Figure S1. Pressure-dependent Raman and IR spectra of $(NH_4)_2SeBr_6$
Figure S2. IR spectra of $(NH_4)_2SeBr_6$ at different pressures and the frequency shifts of these modes.
Figure S3. Refinement lattice parameters and refinement statistics at ambient pressure and 14.26 GPa of $(NH_4)_2SeBr_6$.
Figure S4. Rietveld refinements of $(NH_4)_2SeBr_6$ at different pressures.
Figure S5. The lattice parameters and unit cell volume of $(NH_4)_2SeBr_6$ at different pressures
Figure S6. Schematic illustrations of the band gap evolution of $(NH_4)_2SeBr_6$ upon compression
References

Experimental Section

Sample Preparation and High Pressure Generation. $(NH_4)_2SeBr_6$ was prepared by combing 39.17 mg of NH_4Br (0.4 mmol, Macklin, 99.0%) and 22.19 mg of SeO_2 (0.2mmol, Macklin, 99.9%) in 1mL of 57% (w/w) HBr, which was stirred for 30 min. This solution was slowly cooled to room temperature and placed in a freezer overnight. The resulted yellow powder was washed three times with hydrobromic acid and vacuum-dried overnight.[1] High pressure experiments were performed with a symmetric diamond anvil cell (DAC). The culet diameter of the diamond anvils was 400 μm. The sample was loaded into a 150 μm diameter hole of the T301 steel gasket, which was preindented to a thickness of 40 μm. A small ruby chip was inserted into the sample compartment for in situ pressure calibration, utilizing the R1 ruby fluorescence method. Silicon oil was utilized as the pressure transmitting medium (PTM) for optical absorption and XRD experiments, while the argon was employed as PTM for Raman measurements.

In situ High Pressure Measurements. High pressure angle-dispersive X-ray diffraction (ADXRD) experiments with a wavelength of 0.6199 Å beam were carried out at BL15U1, Shanghai Synchrotron Radiation Facility (SSRF), China. CeO_2 was used as the standard sample to do the calibration. The collected 2D images were integrated on the basis of FIT2D program, yielding 1D intensity versus diffraction angle 2-theta patterns. Refinements of XRD patterns were accomplished by using the Reflex module in Materials Studio Software.

High-pressure absorption spectra were measured in the exciton absorption band region by a deuterium-halogen light source and recorded with an optical fiber spectrometer (Ocean Optics, QE65000). The transmission spectrum of silicon oil around the sample was subtracted as the background.

High-pressure Raman spectra were recorded using a spectrometer equipped with liquid nitrogen cooled CCD (iHR 550, Symphony II, Horiba Jobin Yvon). A 532 nm single-mode DPSS laser was utilized to excite the sample, and the output power was 10 mW. The resolution of the system was 1 cm^{-1}.

High-pressure IR measurements were conducted using a Nicolet iN10 FT-IR micro-spectrometer.

Computational Methodology. The first-principles calculations based on density functional theory (DFT) were performed using the plane-wave pseudopotential as implemented in the VASP code. The electron-core interactions were described with the frozen-core projector-augmented wave

pseudopotentials. The generalized gradient approximation formulated by Perdew, Burke, and Ernzerhof (PBE) as the exchange correlation functional with cutoff energies of 400 eV was chosen in all of our calculations. A reciprocal space sampling with a $6 \times 6 \times 6$ and $8 \times 8 \times 6$ *k*-points is set in the Brillouin zone for cubic and tetragonal phase. The total energy convergence criteria of 1.0×10^{-5} eV and the force on each atom converge to 0.01 eV $Å^{-1}$ while optimizing the geometric structure.

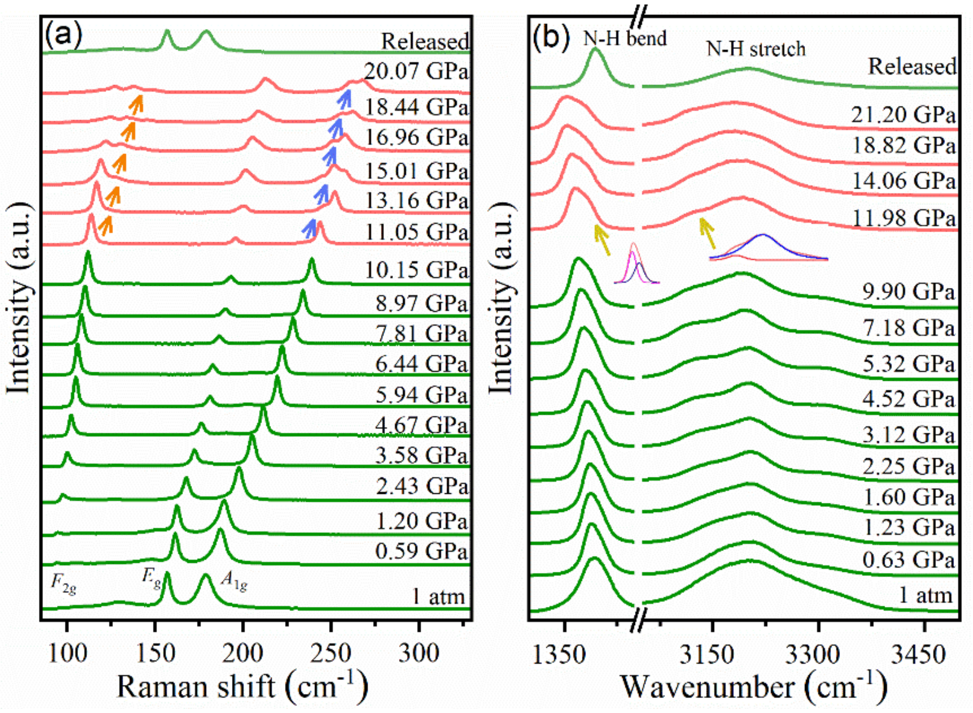

Figure S1. Pressure-dependent Raman (a) and IR spectra (b) of $(NH_4)_2SeBr_6$. The interaction between halides and organic cation inside the inorganic cage is well known to influence the lattice parameters and structural memory effect. To effectively explore the interplay between the NH_4^+ group and the electronegative Br atoms, we further perform *in situ* high pressure Raman and IR experiments. Under ambient condition, only three bands are observed. All bands pertain to Se-Br vibrations in the $[SeBr_6]^{2-}$ octahedra. The strong peaks at 157 (E_g) and 179 cm^{-1} (A_{1g}) can be assigned to the symmetric Br-Se-Br stretching and asymmetric stretching modes, respectively. The low frequency peak at 92 cm^{-1} (F_{2g}) can be assigned to the Se-Br asymmetric bending modes.[2, 3] Moreover, all vibrational modes are found to shift to higher frequency with increased pressure, indicating the hardening of the chemical bonding during compression. Above 0.59 GPa, the intensity of the F_{2g} mode increases gradually. With increased pressure up to approximately 11.05 GPa, except for the frequency shifts, additional splitting of the Se-Br bending and stretching modes is also observed, as indicated by the arrows. Such observation can be assigned to the rotation and distortion of $[SeBr_6]^{2-}$ octahedra, directly supporting the phase transition of $(NH_4)_2SeBr_6$ from cubic to tetragonal phase. Except for the phase transition, all Raman bands exhibit normal blue shift. The

IR spectra of $(NH_4)_2SeBr_6$ show one broad band in 1394 cm^{-1} and three discernable bands between 3050 and 3400 cm^{-1}, which can be assigned to the N-H bending mode and N-H stretching mode, respectively.[4]

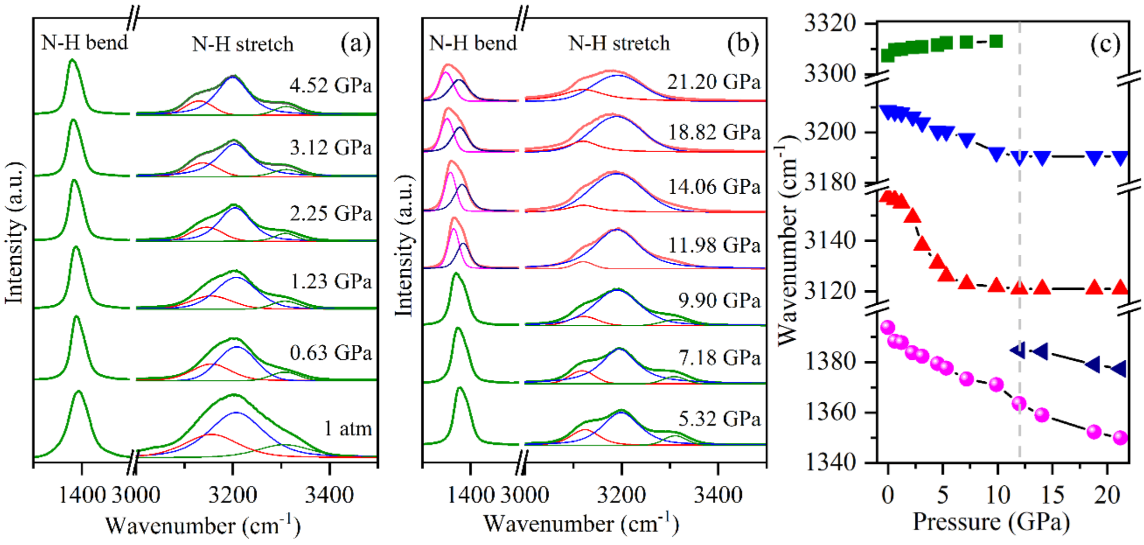

Figure S2. (a-b) IR spectra of $(NH_4)_2SeBr_6$ at different pressures and (c) the frequency shifts of these modes. Most of IR modes are found to continuously red shift at different rates. The red shift suggests that the N-H distance is extended, resulting in the strengthening of N-H…Br hydrogen bond under high pressure. With increased pressure, the N-H bending mode split into two modes at 11.98 GPa, leading to a distortion and rotation angle for the $[SeBr_6]^{2-}$ octahedron due to the hydrogen bond interaction.[5, 6] Hence, the organic cation has a strong influence on the rotation and distortion of $[SeBr_6]^{2-}$ octahedron and in turn affects the stability of $(NH_4)_2SeBr_6$. Upon releasing the pressure, the flexible organic cations NH_4^+ serve as templates in $[SeBr_6]^{2-}$ octahedral frameworks, resulting in the structural memory effect in reversible pressure-induced phase transition.

	1 atm	14.26 GPa
temperature/K	293(2)	293(2)
crystal system	cubic	tetragonal
space group	*Fm-3m*	*P*42
a / Å	10.4826	7.0046
b / Å	10.4826	7.0046
c / Å	10.4826	10.1299
$\alpha=\beta=\gamma$ /°	90	90
Volume/ Å^3	1151.91	497.02
θ range /°	5-20	5.5-17.8
Wavelength / Å	0.6199	0.6199
R_{wp} / %[6]	2.23	0.75
R_p / %[6]	1.48	0.59

Figure S3. Refinement lattice parameters and refinement statistics at ambient pressure and 14.26 GPa for $(NH_4)_2SeBr_6$ crystal. The XRD pattern under ambient condition reveals that the initial crystal structure is the pure cubic phase with space group *Fm*-3*m*. The cubic structure of $(NH_4)_2SeBr_6$ crystal is used as a starting pattern, and the refinement results in cell parameter a = 10.4826 Å. And a tetragonal with space group *P*42 (a = 7.0046 Å, c = 10.1299 Å) is used to fit the split diffraction peaks at 14.26 GPa.

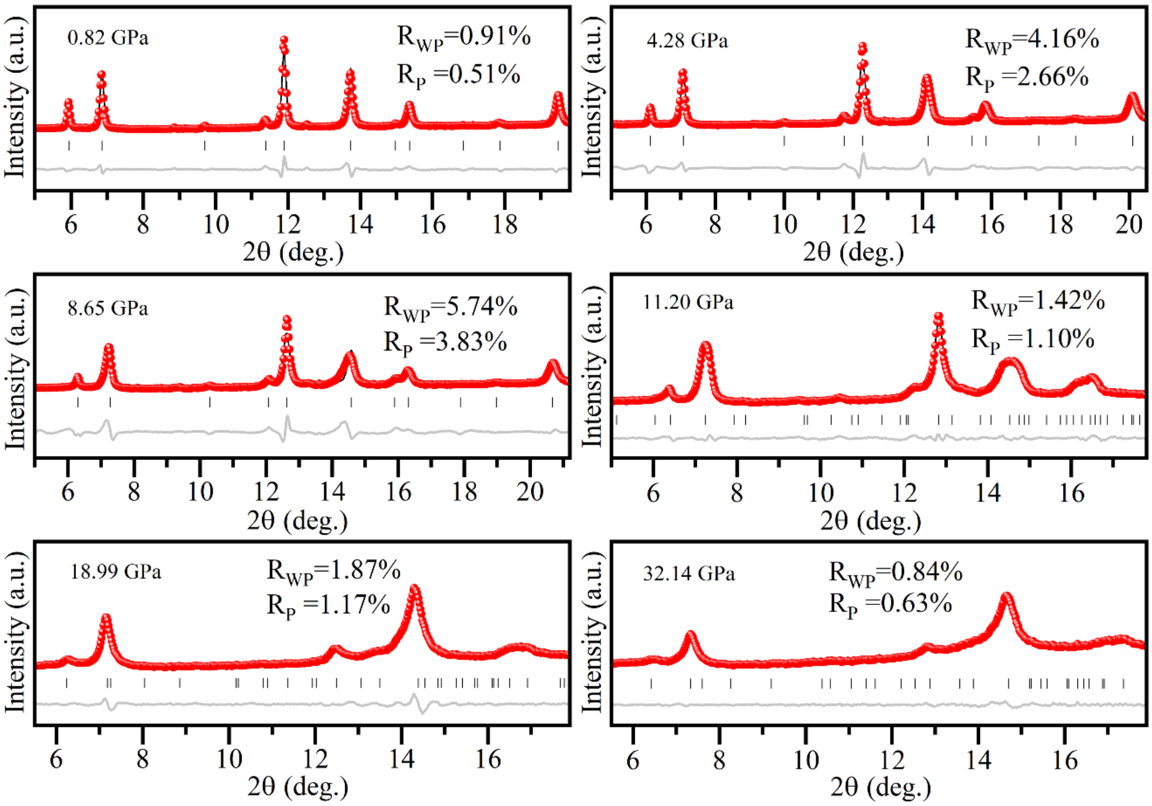

Figure S4. Rietveld refinements of $(NH_4)_2SeBr_6$ at different pressures.

	Pressure (GPa)	a (Å)	b (Å)	c (Å)	V (Å^3)
	**0**	10.4826	10.4826	10.4826	1151.91
	**0.33**	10.4525	10.4525	10.4525	1141.98
	**0.82**	10.3671	10.3671	10.3671	1114.23
	**1.26**	10.3208	10.3208	10.3208	1099.38
**Cubic**	**2.17**	10.1994	10.1994	10.1994	1061.03
**(Fm-3m)**	**3.36**	10.1129	10.1129	10.1129	1034.27
	**4.28**	10.0510	10.0510	10.0510	1015.40
	**5.20**	9.9755	9.9755	9.9755	992.67
	**6.96**	9.8708	9.8708	9.8708	961.73
	**7.83**	9.7824	9.7824	9.7824	937.03
	**8.65**	9.7706	9.7706	9.7706	932.75
	**10.43**	9.6998	9.6998	9.6998	912.63
	**14.26**	7.0046	7.0046	10.1299	497.02
	**15.95**	7.0020	7.0020	10.0610	493.27
**Tetragonal**	**18.99**	7.0010	7.0010	9.7979	480.24
**(P42)**	**21.57**	6.9799	6.9799	9.6396	469.64
	**25.78**	6.9776	6.9776	9.5369	464.33
	**32.14**	6.8540	6.8540	9.3587	439.65

Figure S5. The lattice parameters and unit cell volume of $(NH_4)_2SeBr_6$ at different pressures.

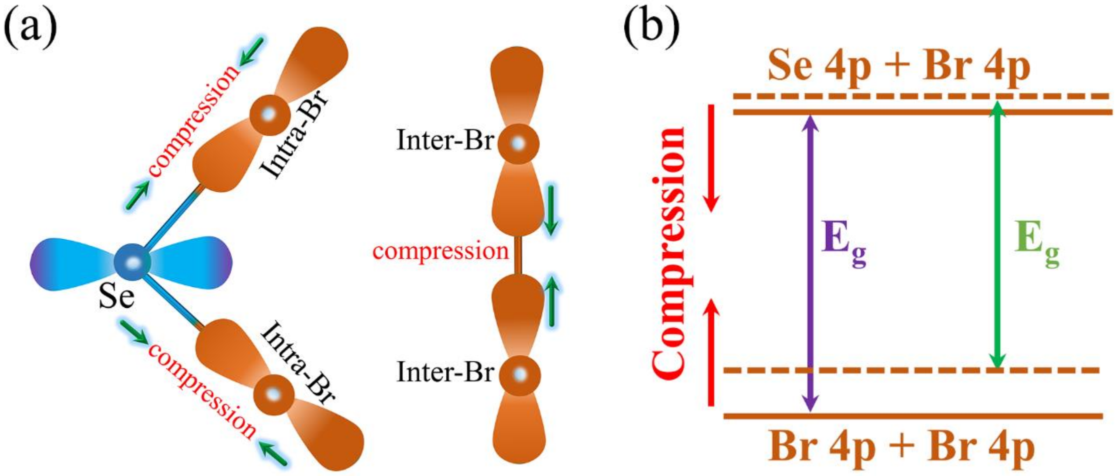

Figure S6. Schematic illustrations of the band gap evolution of $(NH_4)_2SeBr_6$ upon compression. The bandgap of $(NH_4)_2SeBr_6$ is highly variable based on the overlap of electronic wave functions between metal Se and halide Br ions. As for the Se-Br bond lengths and the intra-octahedral Br−Br distances, they are contracted as the $[SeBr_6]^{2-}$ octahedra compression are contracted with the increase in pressure, resulting in a broadening of valence and conduction bands due to their antibonding characteristics. However, the shortest Br-Br bonds of the cubic phase will definitely change from the intra- to inter- octahedra under mild pressure, which mainly influence the band gap of defect perovskites. Given the stiffness of the octahedra, the Se-Br bond is considerably stronger than the Br-Br bond either on the surface of the octahedra or between the isolated octahedra. Hence, VBM rises up greater that of the CBM, and consequently the bandgap is narrowed.

References:

[1] W. Abriel, in *Zeitschrift für Naturforschung B*, **1987**, 42, 415.

[2] P. J. Hendra, Z. Jović, *J. Chem. Soc. A*, **1968**, 600.

[3] E. R. Clark, M. A. Al-Turaihi, *J. Organomet. Chem.* **1977**, 124, 391.

[4] B. V. S Murthy, K. P. Ramesh, J. Ramakrishna, *Phase transit.* **1994**, 46, 229.

[5] L. Wang, K. Wang, B. Zou, *J. Phys. Chem. Lett.* **2016**, 7, 2556.

[6] D. Liu, W. Lei, K. Wang, G. Bao, F. Li, J. Hao, B. Liu, T. Cui, Q. Cui, G. Zou, *J. Phys. Chem. B* **2009**, 113, 7430.
